# Protective effects of fermented goat milk on genomic stability, oxidative stress and inflammatory signalling in testis during anaemia recovery

**DOI:** 10.1038/s41598-018-37649-6

**Published:** 2019-02-19

**Authors:** Jorge Moreno-Fernandez, María J. M. Alférez, Inmaculada López-Aliaga, Javier Diaz-Castro

**Affiliations:** 1 Department of Physiology, University of Granada, Granada, Spain; 2 Institute of Nutrition and Food Technology “José Mataix Verdú”, University of Granada, Granada, Spain

**Keywords:** Reproductive disorders, Urogenital reproductive disorders

## Abstract

Oxidative stress is a harmful factor for male reproductive function, and a major cause of infertility. On the other hand, fermented goat milk has positive effects on anemia recovery and mineral metabolism. This study evaluated the effect of feeding rats with fermented milks during anaemia recovery on molecular mechanisms linked to oxidative stress and inflammatory signalling in rats reproductive system. Forty male Wistar rats were placed on a pre-experimental period of 40 days (control group, receiving normal-Fe diet and Fe-deficient group, receiving low-Fe diet). Lately, rats were fed with fermented goat or cow milk-based diets during 30 days. After feeding the fermented milks, Total antioxidant status (TAS) and non-esterified fatty acids (NEFA) increased and 8-hydroxy-2’-deoxyguanosine (8-OHdG), 15-F2t-isoprostanes and thiobarbituric acid reactive substances (TBARS) decreased in testis. DNA oxidative damage in testis germ cells was lower with fermented goat milk. Fermented goat milk reduced IL-6 and TNF-α in control animals, increasing INF-γ in control and anaemic rats. NRF2 and PGC-1α protein levels increased in testis after fermented goat milk consumption in control and anaemic rats. Fermented goat milk also increased TAS and decreased oxidative damage, protecting the main testis cell bioconstituents (lipids, proteins, DNA, prostaglandins) from oxidative damage and reduced inflammatory activity, preventing injuries to testis germinal epithelium. Fermented goat milk enhanced lipolysis, fatty acids degradation and immune response, attenuating inflammatory signalling, representing a positive growth advantage for testicular cells.

## Introduction

Oxidative stress is a really damaging factor for male reproductive function, and it is considered a cause of male infertility due to its deleterious effects on the developing germ cells and sperm function^1^, inducing male infertility. Therefore, male reproductive tract homeostasis, spermatogenesis and sperm function require protection from oxidative stress^2^. In addition, recent studies have shown that inflammatory signalling plays a key role in the testis regulating endocrine function, spermatogenesis and functional development of male reproductive organs^3^.

On the other hand, the available data suggests that sperm anomalies and changes in morphology are associated with higher generation of reactive oxygen species (ROS) and reduced antioxidant status, i.e. oxidative stress^4^. Nuclear factor (erythroid-derived 2)-like 2 (NRF2) plays a main role in antioxidant defence, protecting male reproductive tract from oxidative stress. Deficiency in NRF2 function has deleterious effects in Sertoli and germ cells, during spermatogenesis and also in sperm cells during epidydimal maturation^5^.

It is also known that antioxidants induce a beneficial role for male germ cells, protecting them from apoptosis induced by damaging factors, such as reactive oxygen or nitrogen species^6^. In addition, it has been previously reported that melatonin (a powerful endogenous antioxidant) caused a significant decrease in oxidative stress intensity. This antioxidant is capable of decrease lipid peroxidation, causing a significant decrease in acid DNAase activity, and reducing amounts of apoptosis in the testicular tissue exposed to radiations^7^.

On the other hand, the adverse effects of iron deficiency anaemia on male fertility are well known for a long time. Reduced sperm counts, abnormal sperm morphology and reduced sperm motility are to be seen in the ejaculate^8^. Changes in histology, morphology and number have been observed in the germinal epithelium at all stages of the spermatogenic cycle. The reasons for this deleterious changes can be attributed to reduced oxygen supply due to the haematological disorders induced by anaemia^9^; other authors have reported testicular atrophy in rats with hypochromic microcytic anaemia^10^.

Previous findings of our research group showed a higher antioxidant potential, increased melatonin levels and diminished lipid peroxidation in rats consuming fermented goat milk. However, the antioxidant role of fermented goat milk in the testis and reproductive function during anaemia recovery remains to be clarified, which is of high importance considering germ cells specificities and its high sensitivity to oxidative damaging factors. Taking into account all the aforementioned, the aim of the study was to evaluate the effect of fermented cow or goat milks on molecular mechanisms linked to oxidative stress, inflammatory signalling and function in male reproductive system of rats during anaemia recovery.

## Results

### Haematological parameters

After iron depletion during 40 d, hemoglobin (49.39%), red blood cells (43.72%) count and hematocrit decreased (31.95%) (P < 0.001). In contrast, platelets count increased (280%) (P < 0.001). After supplying the fermented milk-based diets, all the haematological parameters were recovered (Table 2).

**Table 1:** Hematological parameters in control and anemic rats fed either normal-Fe or low-Fe diets (PEP) or fermented cow or goat milk diets^a^.

PEP			EP			
AIN 93G diet			Fermented cow milk based diet		Fermented goat milk based diet	
	Normal-Fe Control group (*n = *20)	Low-Fe Anemic group (*n = *20)	Control group (*n* = 10)	Anemic group (*n* = 10)	Control group (*n* = 10)	Anemic group (*n* = 10)
Total blood						
Hb concentration (g/L)	121.421 ± 2.652	59.974 ± 2.472*	127.971 ± 2.651	130.251 ± 2.633	132.072 ± 2.680	129.011 ± 2.534
RBCs (10^12^/L)	6.980 ± 0.221	3.052 ± 0.313*	7.140 ± 0.161	7.250 ± 0.251	7.363 ± 0.201	7.545 ± 0.271
Haematocrit (%)	42.091 ± 1.070	13.451 ± 1.471*	39.454 ± 1.310^a^	40.211 ± 1.320^A^	41.881 ± 1.112^b^	42.052 ± 1.101^B^
Platelets (10^9^/L)	757 ± 73.543	2120 ± 117*	939.251 ± 69.673	973.252 ± 67.411	928.001 ± 77.843	941.674 ± 70.652
Serum						
Fe (µg/L)	1225 ± 97.512	617 ± 54.561*	1341 ± 97.381	1230 ± 111.221^A,C^	1359 ± 99.880	1348 ± 95.363^B^
TIBC (µg/L)	2571 ± 153	17641 ± 575*	2841 ± 186	2599 ± 182	2787 ± 183	2753 ± 197
Transferrin saturation (%)	46.951 ± 3.750	4.182 ± 0.501*	44.834 ± 3.840	44.290 ± 4.510	46.327 ± 4.654	46.942 ± 5.115
Ferritin (µg/L)	77.870 ± 2.101	47.981 ± 1.632*	82.353 ± 2.874	81.970 ± 2.370	84.331 ± 2.334	82.342 ± 2.650
Hepcidin, (ng/mL)	16.910 ± 0.621	13.410 ± 0.690*	14.501 ± 0.511	14.581 ± 0.492	16.671 ± 0.515	16.324 ± 0.601

^a^Data are shown as the mean values ± SEM.

Hb, hemoglobin; RBCs, red blood cells; TIBC, total Fe-binding capacity.

*Significantly different from the control group (P < 0.001, Student’s t test).

^a,b^Mean values within a row and within control groups with different superscript lowercase letters differ (*P* < 0.05) by Student’s *t* test.

^A,B^Mean values within a row and within anemic groups with different superscript capital letters differ (P < 0.05) by Tukey’s test.

^C^Indicates difference (P < 0.05) for control vs. anemic group within a diet by the Student’s t test.

**Table 2:** Oxidative/antioxidant biomarkers in testes homogenates from control and anemic rats fed for 30 days with fermented cow or goat milk-based diets with normal or supplemented folic acid content^a^.

	Fermented cow milk-based diet		Fermented goat milk-based diet	
	Control group (*n* = 10)	Anemic group (*n* = 10)	Control group (*n* = 10)	Anemic group (*n* = 10)
TAS (mM Trolox eq/ml)	10.936 ± 0.231^a^	10.103 ± 0.385^A^	12.947 ± 0.365^b^	11.519 ± 0.314^B^
15-F2t-isoprostanes (pg/mg lipids)	0.466 ± 0.042^a^	0.344 ± 0.022	0.330 ± 0.043^b^	0.397 ± 0.021
NEFA (mmol/L)	0.632 ± 0.074^a^	0.637 ± 0.100^A^	0.799 ± 0.085^b^	1.105 ± 0.066^B,C^
TBARS (nmol/mg protein)	0.392 ± 0.028^a^	0.286 ± 0.052^A^	0.270 ± 0.040^b^	0.190 ± 0.025^B^
PC (nmol/mg protein)	1.390 ± 0.094	1.250 ± 0.187	1.164 ± 0.124	1.058 ± 0.077
8-OHdG (ng/ml)	559.574 ± 35.157^a^	455.954 ± 23.312^A,C^	479.141 ± 22.576^b^	516.843 ± 18.954^B^
AOPP (mmol/mg protein)	135.585 ± 10.353	150.989 ± 9.865^A^	121.862 ± 11.925	76.983 ± 11.289^B,C^

^a^Data are shown as the mean ± SEM.

TAS, total antioxidant status; NEFA, non strerifed fatty acids; TBARS, Thiobarbituric acid-reactive substances; PC, Protein carbonyl; 8-OHdG, 8-hidroxy-2′-deoxyguanosine guanosine; AOPP, advanced oxidation protein products.

^a,b^Mean values within a row and within control groups with different superscript lowercase letters differ (*P* < 0.05) by Tukey’s test.

^A,B^Mean values within a row and within anemic groups with different superscript capital letters differ (*P* < 0.05) by Tukey’s test.

^C^Indicates difference *(P* < 0.05) for control *vs*. anemic group within a diet by the Student’s *t* test.

### Oxidative stress

With regard to the oxidative stress-mediated damage to the main biomolecules, Table 3 shows that after 30 days of feeding the fermented milk-based diets, TAS was higher in both groups of animals (16% for control and 13% for anaemic) fed fermented goat milk with respect to fermented cow milk (P < 0.05). Testes 8-OHdG, 15-F2t-isoprostanes and TBARS concentrations were lower in control animals fed fermented goat milk (16%, P < 0.01; 41%, P < 0.05 and 45%, P < 0.05 respectively). NEFA concentration was higher in control (21%, P < 0.05) and anaemic rats (43%, P < 0.001) fed with fermented goat milk. No differences were found in protein carbonyl (PC) and advanced oxidation protein products (AOPP) decreased dramatically in anaemic animals fed with fermented goat milk (96%, P < 0.001). Anaemia decreased AOPP (58%, P < 0.001) and increased NEFA and 8-OHdG (30%, P < 0.01; 8%, P < 0.01) in animals fed with fermented goat milk.

**Table 3:** DNA damage in testes germ cells from control and anemic rats fed for 30 days with fermented cow or goat milk-based diets^a^.

	Fermented cow milk-based diet		Fermented goat milk-based diet	
	Control group (*n* = 10)	Anemic group (*n* = 10)	Control group (*n* = 10)	Anemic group (*n* = 10)
Tail DNA (%)	42.23 ± 2.95^a^	39.05 ± 2.17^A^	22.59 ± 1.72^b^	15.02 ± 1.49^B,C^
OTM2	1.91 ± 0.29^a^	0.98 ± 0.14^A,C^	0.49 ± 0.06^b^	0.19 ± 0.02^B,C^

^a^Data are shown as the mean ± SEM.

^b^OTM, Olive tail moment.

^a,b^Mean values within a row and within control groups with different superscript lowercase letters differ (*P* < 0.05) by Tukey’s test.

^A,B^Mean values within a row and within anemic groups with different superscript capital letters differ (*P* < 0.05) by Tukey’s test.

^C^Indicates difference *(P* < 0.05) for control *vs*. anemic group within a diet by the Student’s *t* test.

### Genomic stability

**Figure 1:**
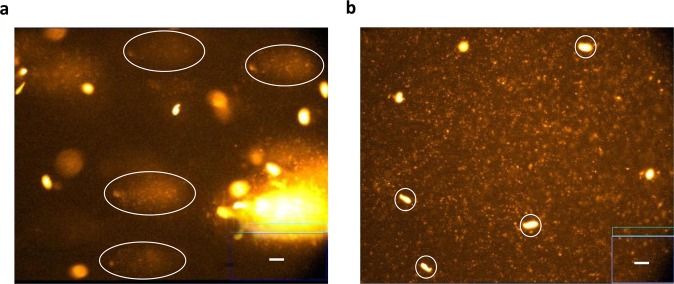
Representative images of germ cells comets, after fermented cow milk based diet **(a)** or fermented goat milk based diet **(b)** consumption. One hundred comets from each gel (scored at random) were scored using computerized image analysis. White bars represent 5 µm. Some representative comets have been circled.

DNA oxidative damage in testis germ cells (Table 4, Fig. 1) was lower when fermented goat milk was supplied, as revealed by the percentage of DNA in tail and olive tail moment (OTM) (P < 0.001), compared with those rats that consumed the fermented cow milk. While anaemia had no effect on tail DNA in animals fed with fermented cow milk, it decreased in animals fed with fermented goat milk (P < 0.001). Anaemia also decreased OTM in animals fed both fermented milks (P < 0.001).

**Table 4:** Pro- and anti- inflammatory cytokines in testes from control and anemic rats fed for 30 days with fermented cow or goat milk-based diets^a^.

	Fermented cow milk-based diet		Fermented goat milk-based diet	
	Control group (*n* = 10)	Anemic group (*n* = 10)	Control group (*n* = 10)	Anemic group (*n* = 10)
INF-γ (pg/mg)	0.207 ± 0.010^a^	0.184 ± 0.022^A^	0.348 ± 0.081^b^	0.276 ± 0.027^B^
IL-6 (pg/mg)	0.026 ± 0.006^a^	0.016 ± 0.002^C^	0.012 ± 0.006^b^	0.016 ± 0.004
TNF-α (pg/mg)	0.090 ± 0.021^a^	0.098 ± 0.009^A^	0.064 ± 0.026^b^	0.068 ± 0.020^B^

^a^Data are shown as the mean ± SEM.

^a,b^Mean values within a row and within control groups with different superscript lowercase letters differ (*P* < 0.05) by Tukey’s test.

^A,B^Mean values within a row and within anemic groups with different superscript capital letters differ (*P* < 0.05) by Tukey’s test.

^C^Indicates difference *(P* < 0.05) for control *vs*. anemic group within a diet by the Student’s *t* test.

### NRF2 and PGC-1α protein expression

**Figure 2:**
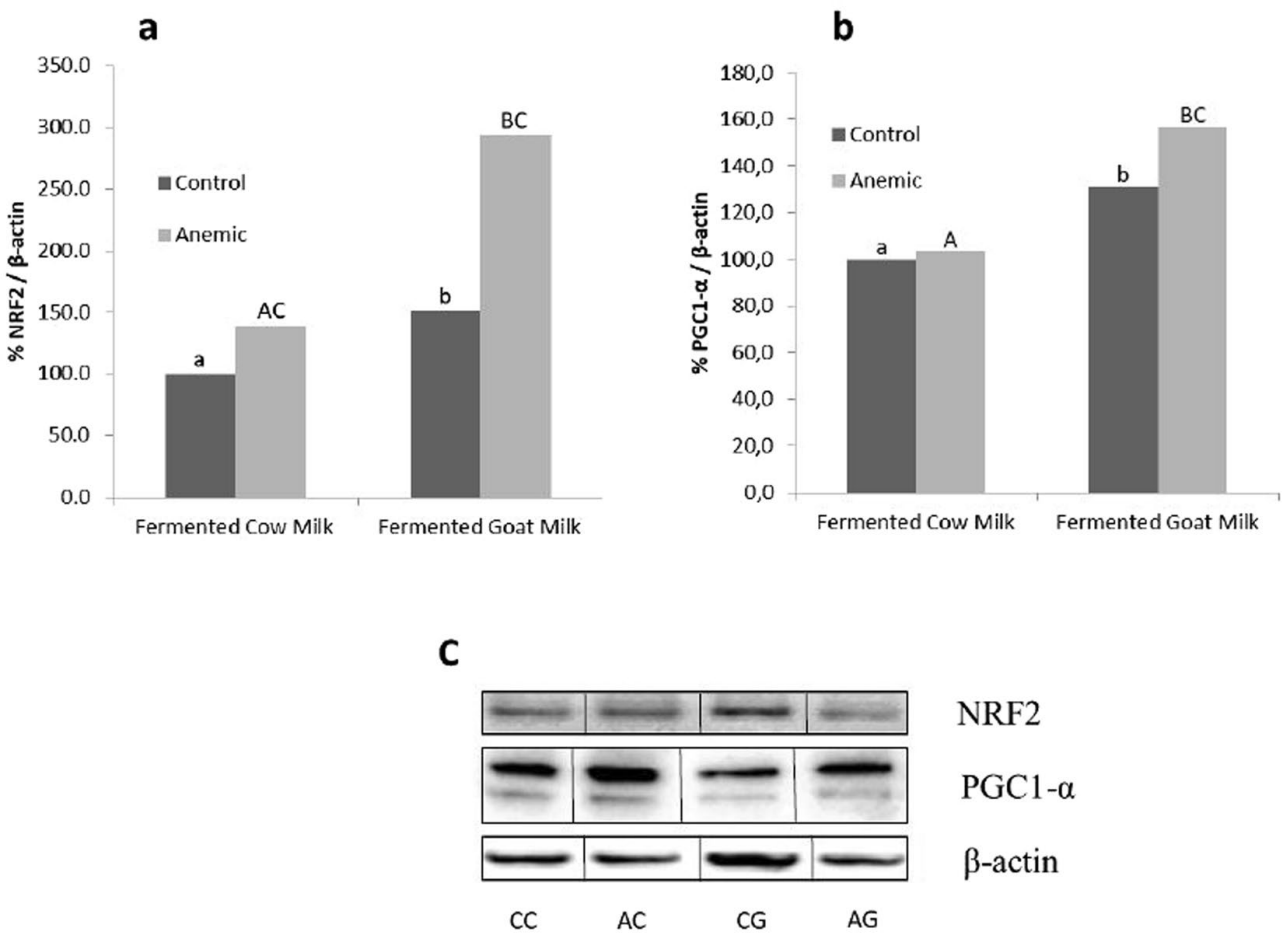
Effect of fermented cow and goat milk in control and anemic rats on testis protein levels of NRF2 (**a**), PGC-1α (**b**) and representative immunoblots (**c**). The full-length western blots are presented in Supplementary Figure S1. Data are means with SEM of 10 animals per group. CC: control cow; AC: anemic cow; CG: control goat; AG: anemic goat. ^a,b^Mean values within a row and within control groups with different superscript lowercase letters differ (*P* < 0.05) by Student’s *t* test. ^A,B^Mean values within a row and within anemic groups with different superscript capital letters differ (*P* < 0.05) by Student’s *t* test. ^C^Indicates difference *(P* < 0.05) for control *vs*. anemic group within a diet by the Tukey’s test.

Protein expression of NRF2 and PGC-1α were analyzed in control and anaemic rats after consumption of fermented cow or goat milk-based diets to explore the homeostatic variations of these oxidative-stress related proteins. The NRF2 expression of control and anaemic rats fed on fermented goat milk was respectively 152% and 293% of the NRF2 expression of rats fed with fermented cow milk. Fe-deficiency increased the NRF2 expression in both groups of animals fed with both types of fermented milk (P < 0.001) Fig. 2a,c). PGC-1α increased in control and anaemic animals fed fermented goat milk (31% and 53% respectively) (P < 0.05; Fig. 2b,c) and increased in response to the iron-deficiency in animals fed fermented goat milk (P < 0.05).

### Inflammatory signaling

Pro- and anti-inflammatory cytokines in testes are shown in Table 5. Fermented goat milk consumption reduced IL-6 in control animals (116%, P < 0.05) and TNF-α in both groups of animals (40% for control, P < 0.05; 44% for anemic, P < 0.05), while increased INF-γ in both experimental groups (41%, P < 0.05 for control; 34%, P < 0.05 for anemic).

**Table 5:** Composition of the experimental diets.

Component	g/Kg diet
**Pre-experimental period, Standard (non-milk) diet** ^**a**^	
Casein	200
Lactose	0
Fat (virgin olive oil)	100
Wheat starch	500
Constant ingredients^b^	200
**Experimental period**	
**Fermented cow milk-based diet** ^**c**^	
Protein	205
Lactose	295
Fat	100
Wheat starch	200
Constant ingredients^b^	200
**Fermented goat milk-based diet** ^**c**^	
Protein	206
Lactose	291
Fat	100
Wheat starch	203
Constant ingredients^b^	200

^a^The diets were prepared according to the recommendations of the AIN-93G for control rats (45 mg Fe/Kg diet) (Reeves *et al*., 1993), or with low Fe content (5 mg Fe/Kg diet) (Pallarés *et al*., 1993), for anemic groups.

^b^The constant ingredients consisted of (g/Kg diet): fibre (micronized cellulose) 50, sucrose 100, choline chloride 2.5, L-cystine 2.5, mineral premix 35, vitamin premix 10.

^c^Specific vitamin and mineral premixes supplements for fermented cow and goat milk-based diets were formulated taking into account the mineral and vitamin contents of the fermented milk powder supplied in order to meet the recommendations of the AIN-93G for normal-Fe diets (45 mg /Kg diet) (Reeves *et al*., 1993).

## Discussion

To the best of our knowledge, there are no data available on the effects of fermented milks on testicular oxidative stress in rats. Therefore, the present investigation was undertaken to evaluate the *in vivo* effects of fermented milk consumption on normal testis function during anaemia recovery in terms of oxidative stress and inflammatory signalling.

Mammalian spermatogenesis is a complex biological process occurring in the seminiferous tubules in the testis. Spermatogenesis comprises a delicate balance between cell proliferation, differentiation and apoptosis^11^. In most mammals, this process proceeds with blood and O_2_ supply that is fairly independent from changes in other vascular beds in the body. Despite this well-controlled local environment in the testicular tissues, hypoxia induces adverse effects on testicular cell function and spermatogenesis. This condition may lead to male subfertility or infertility^12^. In case of generalized hypoxia, as occurs in severe Fe-deficiency, the blood flow is predominantly distributed to the vital organs (brain and heart) leading to local decrease in testicular blood flow and affecting testicular function^13^. The observation of testicular atrophy after hypochromic microcytic anaemia has been explained as sequels of the hypoxaemia due to the haematological disorders^10^. In addition, Sertoli and Leydig cells are important sources of ferritin which acts as a source of Fe for the developing spermatozoa, while providing an extra layer of protection to the testicular tissue playing a critical role in the synthesis of nucleic acids and proteins, electron transport, cellular respiration, proliferation and differentiation, all of which are intimately related to spermatogenesis and spermatozoa metabolism (Wise *et al*., 2003), therefore in anemia situation, this layer and immature spermatozoa can be seriously impaired. The inclusion of the blood parameters in the current study is important because haemoglobin has a key role on oxygen transport, and any significant changes in this protein have a significant impact on the oxygen supply, including in the testes, especially under hypoxic conditions. Therefore, haemoglobin concentration and haematological status determine the oxygen concentration of the blood^12^. At the end of the EP, after feeding the animals with fermented cow or goat milk diets, all the haematological parameters were recovered, indicating that a normal Fe status was established again in all the animals and the decrease in testicular blood flow was restored.

Under physiological conditions, the immunosuppressive testicular microenvironment protects germinal cells from being attacked by the immune system. However, under inflammatory conditions, this tolerance is disrupted and immune cells and their mediators respond to germinal cell self-antigens, inducing damage to the germinal epithelium^14^. These mediators include soluble proteins and bioactive small molecules that are either constitutively present in biological fluids or that are released from activated cells (including cytokines that regulate the function of other cells, chemokines that attract inflammatory leukocytes, lipid mediators of inflammation, and reactive oxygen species) (Chaplin, 2009). Thus, we set out to study testicular damage by examining antioxidant/oxidative processes and the link with the inflammatory signaling as it is currently regarded as one of the most important causes of impaired testicular function.

Fermented goat milk consumption leads to reproductive protection by decreasing oxidative stress, increasing antioxidant levels in testes, reducing the damage to the main biomolecules^15^. Reactive oxygen and nitrogen species interacts with lipids present on biological membranes, altering their structure, function and permeability, being MDA the last product of decomposition of polyunsaturated fat acids in cell membranes. Testicular membranes are rich in polyenoic fatty acids, which are particularly vulnerable against oxidative deterioration. In addition, oxidation protein oxidation can also lead to breakage of cells integrity and loss of function. These actions are referring to be etiology of reproductive toxicology^16^. The damage induced by reactive oxygen species in the germinal and testis cell membrane may be the cause of death, abnormality and motility loss^17^.

Lipid peroxidation causes loss of function in membranes, death cell through necrosis and ultimately testicular dysfunction^18,19^. The oxidative stress causes damage on spermatological parameters^20^. Fermented goat milk consumption significantly reduced the oxidative stress by decreasing lipid peroxidation and increasing antioxidant system activities. Because fermented goat milk increased the testicular antioxidant status without peroxidative damage, this fact implies that the testis antioxidant defense system effectively protects from the oxidative stress in this experimental group. It seems that the increase in the antioxidant system causes more rapid conversion of free radicals preventing subsequent peroxidative damage. This is further supported by the lower peroxidation adducts concentration in the rat testis in animals consuming fermented goat milk. Furthermore, it is believed that inducing antioxidant system in response to an oxidative insult can be protective as a way of developing ‘tolerance’ to a subsequent larger insult^21^. Previous studies of our research group have shown that goat milk has a better lipidic quality compared with cow milk, improving the nutritive utilization of fat and diminishing plasma total cholesterol^22,23^. The better nutritive utilization of goat milk fat, the healthy lipid plasma profile and the improvement in antioxidant systems and reduced oxidative stress recorded with goat milk consumption, reduces lipid peroxidation avoiding the oxidative damage to the lipids^22^. Additionally, as previously reported^24^, fermented goat milk has higher amounts of tyrosine, methionine, histidine, lysine, and tryptophan, which are capable of chelating pro-oxidative metal ions, inhibiting lipid (TBARS) and AOPP.

Male infertility is also associated with increased DNA damage in germinal cells^25,26^. DNA damage can occur independently of standard semen parameters, and increased germinal cells fragmentation is correlated with infertility^27^. For this reason, measurement of DNA damage in testes germinal cells is very important to evaluate the reproductive effects of fermented milks during Fe repletion. In this assay, isolated germinal cells with high DNA strand breaks show intense comet tail and increased comet tail length due to the increased DNA fragmentation^28^. Germinal cells DNA damage decreased in animals fed fermented goat milk. As previously reported^29^, fermented goat milk consumption increases plasma melatonin levels, fact that could explain the increase in TAS recorded in the current study, however there are other nutritional characteristics of goat milk that contributes to the action of melatonin in ameliorating oxidative stress due to modulation of antioxidant defenses. In addition, habitual goat milk consumption has positive effects on enzymatic antioxidant defence (catalase, superoxide dismutase and glutathione peroxidase), limiting the generation of free radicals^15^. In addition, fermented goat milk has higher amounts of antioxidant minerals (especially Mg, Zn and Se)^24^, which play a positive role in antioxidant status^23,30^, enhancing genomic stability, acting as cofactors in DNA double-strand break repair and higher amounts of vitamin A, which can be incorporated into plasma and contributing to antioxidative capacity^31^. Finally, goat milk has bioactive peptides^24^, which have also some antioxidative properties, explaining the lower rate of 8-OHdG and 15Ft-isoprostanes achieved in the current study with fermented goat milk. As previously mentioned, spermatogenic process requires blood and O_2_ supply that is independent of changes in other vascular beds. Despite this, hypoxia result in adverse effects on testicular cell function and spermatogenesis^12^. However, taking into account that OTM was reduced in anaemia situation, we can assume that because the oxygen supply is restored during the anaemia recovery and the Fe trafficking in the organism is mainly distributed to haematopoietic organs. In situation of deficiency, DNA strand breaks are reduced, because the adequate antioxidant status in testis, together with an insufficient amount of Fe available, would exert a protective effect, avoiding the Fe-catalyzed generation of oxygen radicals via Fenton and Haber-Weiss chemistries, explaining and supporting our findings regarding biomolecules stability and oxidation^32^.

In physiological conditions, NRF2 is expressed at low level in testis and resides mainly in cell cytoplasm, where it is promoted to degradation via ubiquitination by a repressor protein^33^. Under oxidative stress or through NRF2 activators such as xenobiotics, electrophiles or phytochemicals, NRF2 translocates to the nucleus and then expression of genes with antioxidant response element (ARE) is activated^34^. The protective mechanism activated by NRF2 involves induction of antioxidant defense. Our study showed that fermented goat milk increased TAS in testis, revealing a plausible molecular mechanism for increased antioxidant protection in male reproductive tract during anaemia recovery.

On the other hand, even though Sertoli cells are quiescent, they are metabolically very active, because of their putative metabolic and energetic support to differentiating germ cells. In early studies, it has been previously demonstrated that the oxidation of fatty acids is a major energy source for testicular cells^35^. In the current study, fermented goat milk significantly improved energy availability in testicular cells, as resulted from increased levels of NEFA. This can be explained by the improvement in calcium metabolism. Acute effects of calcium may result from an increase in lipolysis, raising NEFA availability and reduced inhibition of carnitine and long-chain fatty acid esterification. In this sense, as we have previously reported, goat milk increases calcium bioavailability^36^, due to the higher content of vitamin D, which favors calcium energy-dependent transcellular saturable transport, therefore this can be one reason explaining the effect of fermented goat milk effect enhancing lipolysis and levels of NEFA, indicating an improvement in fatty acids degradation and representing a positive growth advantage of testicular cells.

Regulation of metabolic genes in testis is thought to involve the peroxisome-proliferator-activated receptor-γ coactivator-1α (PGC-1α). This protein bind to and coactivates transcription factors, estrogen-related receptor, and nuclear respiratory factor-1, and -2 resulting in enhanced expression of mitochondrial respiratory chain enzymes and genes involved in fatty acid oxidation and mitochondrial biogenesis^37^. In our study, fermented goat milk consumption induced an over-expression of PGC-1α in both groups of animals. Mitochondria are the main sites for fat metabolism and are also the major source of reactive oxygen species, a byproduct of the mitochondrial electron transport chain. However, in the current study, the over-expression of PGC-1α does not imply an increase in oxidative stress, but enhanced lipolysis, and fatty acids degradation which can be correlated with NEFA levels and representing, once more a positive metabolic advantage for testicular cells.

Inflammatory pathways play a role in normal testicular development and represent an important mode of action for testes toxicity. Inflammatory cytokines are produced by multiple testis cell types including macrophages, Sertoli and Leydig cells. Cytokine signalling plays an important regulatory role in testes development and function^38^. Although the testes are considered to be “immuneprivileged” due to their ability to tolerate autoantigens that are expressed by germ cells, inflammation can occur due to a number of factors like exposure to toxic compounds^39^. Fermented cow milk consumption led to an increase in levels of pro-inflammatory markers IL-6, and TNF-α and a decrease in IFN-γ compared with fermented goat milk. It has been previously reported that dietary selenium enhances host systemic immune response^40^ and the levels of these pro-inflammatory cytokines, which have different synthesis locations and functions, decrease by dietary Se^41^. As previously mentioned, fermented goat milk has higher amounts of Se^24^, this fact together with the improvement in antioxidant status, could enhance immune response, attenuating inflammatory signalling mediated by IL-6, and TNFα in the testicular cells.

The limitation of this study was the lack of more fresh biological samples to get better and more representative Comet assay figures. Although the software to process and quantification this assay is powerful enough and has allowed to obtain robust results, a higher amount of sample would allow us to obtain better figures.

In conclusion this study provides new insights into the development of local oxidative status and inflammatory processes in testis during anaemia recovery with fermented milks. The study showed that NRF2 protein level in rat testis increased after fermented goat milk consumption, indicating a protective role in male reproductive tract that can be linked to the increased antioxidant status. This effect limits the oxidative damage to the main biomolecules (lipids, protein DNA, prostaglandins), reduces the inflammatory activity, and prevents further injury to the germinal epithelium in testis. In addition, fermented goat milk effect enhances levels of NEFA and PGC-1α, indicating an improvement in fatty acids degradation and representing a positive growth and metabolic advantage for testicular cells. Finally, fermented goat milk consumption improves immune response, attenuating inflammatory signalling mediated by IL-6, and TNFα in the testicular cells. In summary, the knowledge gained from these findings reveals that the inclusion of fermented goat milk in the diet during the recovery of nutritional Fe-deficiency improves gonadal antioxidant status.

## Methods

### Fermentation and dehydration of the milks

Fermented milks were prepared following previously described method by Moreno-Fernandez *et al*.^24^. Both milk types were inoculated with traditional yoghurt starters *Lactobacillus bulgaricus* subsp. *delbrueckii* and *Streptococcus thermophilus* (initial concentration 1 × 10^11^ colony-forming units (CFU) mL^−1^; 10 mL L^−1^ inoculum) and incubated at 37 °C for approximately 24 h. Subsequently, fermented milk samples were subjected to a smooth industrial dehydration process until the final moisture content ranged between 25 and 45 g kg^−1^.

### Animals

Forty male Wistar albino breed rats (6 wks of age and weighing about 175 ± 5 g) were used in this study. Animal assays were carried out in the animal breeding unit of the Centre of Biomedical Research, University of Granada (Spain), in a free pathogens area with high biological safety, sanitary and environmental and rigorously controlled sanitary and environmental conditions. Experimental procedures were approved by the Ethics Committee of Animal Experimentation at the University of Granada in strict agreement with the guidelines and regulations about animal welfare and experimentation set by the European Commision guidelines (Declaration of Helsinki; Directive 2010/63/EU).

Rats were allocated randomly in individual, ventilated, thermoregulated cages with an automatically controlled temperature (22–23 °C), humidity (55–65%) and a 12-hour light-dark cycle (9:00 to 21:00) during the course of the study. Diet intake was controlled, pair feeding all the animals (80% of the average intake) and bidistilled water was available *ad libitum*.

### Experimental design

During the pre-experimental period (PEP) (N = 40) rats were divided into two groups: the control group receiving the AIN 93 G diet with normal-Fe diet (44.8 mg/kg by analysis)^42^, and the anaemic group receiving the same diet, but with a low-Fe content (6.1 mg/kg by analysis), induced experimentally during 40 d by a method developed previously by our research group^43^. In this period the Fe restriction in the diet was severe enough to impair haematological status and the ferropenic anaemia was induced.

When the induction of the anaemia period finalized (d 40 of the study), the animals subsequently started the experimental period (EP) in which the control and anaemic groups were fed for 30 days with fermented cow milk or fermented goat milk-based diet, with normal-Fe content (45 mg/kg), prepared with fermented cow (Holstein breed) or fermented goat milk (Murciano-granadina breed) powder (20% of protein and 10% of fat). The Fe content in these diets by analysis was 42.7 mg/kg in fermented cow milk-based diet and 43.5 mg/kg in goat milk-based diet. (Table 1).

At the end of the EP (d 70 of the study), animals were anesthetized intraperitoneally with sodium pentobarbital (Sigma-Aldrich Co., St. Louis, MO), totally bled out by cannulation of the aorta. Blood aliquots with EDTA were analysed to measure the haematological parameters and the rest of the blood was centrifuged (1500 × *g*, 4 °C, 15 min) without anticoagulant to separate the red blood cells from the serum and to evaluate iron, total iron binding capacity, ferritin and hepcidin, Both testes were extracted and weighed. The right testicle was split into two portions: one of them was homogenized in a buffer solution of potassium phosphate (pH 7.4) and centrifuged at 2000 × *g*/15 min at 4 °C, preserving these supernatant fractions at −80 °C for further analyses of TAS, 8-OHdG, 15-F2t-isoprostanes, NEFA, TBARS and PC. The remaining portion of the right testicle was washed repeatedly with ice-cold deionized water, snap frozen in liquid nitrogen and immediately stored at −80 °C. This aliquot was used for protein extraction, analysis of advanced oxidation protein products, cytokines and western blotting (NRF2 and PGC-1α). The left testicle was used for germinal cell extraction and comet assay. Protein content was estimated by the Lowry method^44^ using a bovine serum albumin as a standard.

### Haematological test

Hb concentration (g/L), red blood cells (RBCs) (10^12^/L), haematocrit (%) and platelets (10^9^/L) of fresh blood samples were measured using an automated hematology analyzer Sysmex K-1000D (Sysmex, Tokyo, Japan).

### Serum iron, total iron binding capacity (TIBC) and transferrin saturation

To calculate the rate of transferrin saturation (%), serum iron concentration (µg/L) and TIBC (µg/L) were determined using Sigma Diagnostics Iron and TIBC reagents (Sigma Diagnostics). The absorbance of samples was read at 550 nm on a microplate reader (Bio-Rad Laboratories Inc., Hercules, CA).

### Serum ferritin

Serum ferritin concentration (µg/L) was determined using the Rat Ferritin ELISA Kit (Biovendor Gmbh, Heidelberg, Germany). The absorbance of the reaction was read at 450 nm using a microplate reader (Bio-tek, Vermont, USA). Colour intensity developed was inversely proportional to the concentration of serum ferritin. Measurements in duplicate were used to determine intra-assay variability.

### Serum hepcidin

Hepcidin-25 (ng/mL) was determined using a DRG ELISA Kit (DRG Instruments GmbH, Marburg, Germany). The absorbance was read at 450 nm with a plate reader (Bio-Rad). Measurements in duplicate were used to determine intra-assay variability.

### Total antioxidant status (TAS)

To determine testis homogenate TAS levels (mM Trolox eq/mL), freshly thawed batches of testis homogenate were analysed using TAS Randox kit (Randox laboratories, Ltd, Crumlin, UK). Results were expressed in mM of Trolox equivalents. The linearity of calibration extends to 2.5 mmol/L of Trolox. Measurements in duplicate were used to determine intra-assay variability.

### 15-F2t-isoprostanes

Isoprostanes (pg/mg lipids) in testis homogenate were measured using a commercial kit Enzyme Immunoassay for Isoprostane (Oxford Biomedical Research, Oxford, England). The 15-F2t-Isoprostane competes with 15-F2t-Isoprostane conjugated to horseradish peroxidase (HRP). The HRP activity results in colour development when the substrate is added, with the intensity of the colour proportional to the amount of 15-F2t-Isoprostane-HRP bound and inversely proportional to the amount of unconjugated 15-F2t-Isoprostane in the samples or standards. Plates were read spectrophotometrically (Bio-tek,Vermont, USA) at 450 nm.

### Non-esterified fatty acids (NEFA)

NEFA (mmol/L) were measured using a commercial kit (Randox Laboratories Ltd., Crumlin, UK) following manufacturer´s instructions. Briefly, 50 µl of standards or serum samples were pipetted into eppendorf tubes and mixed with R1and R2 solutions. After 10 minutes, the mixture was measured for the optical density at 550 nm in a spectrophotometer (Bio-tek,Vermont, USA). Measurements in duplicate were used to determine intra-assay variability.

### Thiobarbituric acid reactive substances (TBARS) measurement

Lipid peroxidation was evaluated in testis homogenate by measuring the concentration of TBARS (nmol/mg protein) according to the methods of Yagi^45^ and Ohkawa *et al*.^46^. The reaction product was measured by spectrophotometric analysis (Bio-tek,Vermont, USA) at 532 nm. Measurements in duplicate were used to determine intra-assay variability.

### Protein oxidation (carbonyl groups) measurement

Protein oxidation (nmol/mg protein) in testis homogenate was measured according to a method based on the spectrophotometric detection of the reaction of 2,4-dinitrophenylhydrazine (DNPH) with PC to form protein hydrazones^47^. Each sample of extracted protein was treated with 10 mM DNPH in 2.5 M HCl. PC level was calculated at the maximum absorbance (366 nm) of the DNPH-treated samples. Measurements in duplicate were used to determine intra-assay variability.

### 8-hydroxy-2′-deoxyguanosine (8-OHdG)

8-OHdG in testis homogenate (ng/mL) was measured using a commercial kit (8-OHdG Check, Japan Institute for the Control of Aging, Shizuoka, Japan). To separate interfering substances, the samples were filtered using an ultra-filter (cut off molecular weight 10000 Da) (Amicon Sigma-Aldrich, St Louis, MI). Results were read at 450 nm on a microplate reader (Bio-tek,Vermont, USA). Measurements in duplicate were used to determine intra-assay variability.

### Determination of advanced oxidation protein products (AOPP)

AOPP (nmol/mg protein) was determined in the protein testes extracts using the method described by Witko-Sarsat *et al*.^48^. AOPP results from oxidation of amino acid residues such as tyrosine, leading to the formation of dityrosinecontaining protein cross-linking products detected by spectrophotometry. Measurements in duplicate were used to determine intra-assay variability.

### Analysis of cytokines

Interferon-γ (IFN-γ), Tumour necrosis factor alpha (TNF-α) and Interleukin 6 (IL-6) on protein tissue extracts were determined using the RECYTMAG-65K Milliplex MAP Rat Cytokine/Chemokine Magnetic Bead Panel (Millipore Corporation, Missouri, USA), settings: 50 events per bead, 25 μl sample, gate settings: 8000–15000, time out 60 seconds. Plate was read on a LABScan 100 analyzer (Luminex Corporation, Texas, USA) with xPONENT software for data acquisition. Average values for each set of duplicate samples or standards were within 15% of the mean. Cytokines on protein tissue extracts were determined by comparing the mean of duplicate samples with the standard curve for each assay.

### Western Blotting and Immunocytochemistry

Finely chopped testis samples were obtained using a Potter−Elvehjem homogenizer apparatus on ice, and whole cell proteins were extracted using T-PER tissue extraction reagent (Thermo Scientific Inc., Hanover Park, IL, USA). Protease inhibitor (1:200 dilution; Sigma-Aldrich) was incorporated to avoid protein degradation. Wester blotting procedure was prepared according to the method previously described by Moreno-Fernandez *et al*.^49^. The blots were incubated with NRF2 polyclonal (Abcam, UK; dilution 1:500), PGC-1α monoclonal (Merck., Darmstadt, Germany; dilution 1:1000) and mouse anti-β-actin monoclonal (Abcam, UK; dilution 1:1000) as primary antibodies, in 5% dry milk in TTBS overnight at 4 °C with shaking. β-Actin was used as loading control. The bands were visualized with Luminata forte western HRP substrate (Merck KGaA, Darmstadt, Germany). Signal quantification and recording densitometry of each band were performed with chemiluminescence in ImageQuant LAS 4000 (Fujifilm Life Science Corp., USA). All results were analyzed with ImageJ software. Measurements in duplicate were used to determine intra-assay variability.

### Germ cell preparation and alkaline single cell gel electrophoresis (Comet assay)

One testicle of each rat was carefully decapsulated with a fine forceps avoiding damage to seminiferous tubules. The tubules were then placed in PBS with glucose (0.15 w/v). Decapsulated testes were placed in tubes with collagenase solution (1 mg/ml in PBS) and shaken in water bath at 30 °C for 10 min until testes were dispersed into individual tubules. A solution of trypsin 0.25% (w/v) in PBS was added. The non-digested tissue was removed and the testicular germ cells were used for comet assay as previously described^32^. DNA strand breaks were detected using the alkaline comet assay, or single-cell gel electrophoresis. Isolated germ cells were resuspended in 85 µL of 1% low–melting-point agarose (Invitrogen, Scotland, United Kingdom; w/v, in phosphate buffered saline, 37 °C, pH 7.4) and pipetted onto a frosted-glass microscope slide precoated with 85 µL of 1% high–melting-point agarose (Invitrogen; w/v, in phosphate buffered saline, pH 7.4). The agarose was allowed to set for 5–6 min at 4 °C and the slide was incubated for 1 h in a lysis solution (2.5 mol/L of NaCl, 10 mmol/L of Tris, 100 mmol/L of Na_2_EDTA, NaOH to pH 10, and 1% [v/v] Triton X-100; Sigma Diagnostics). The slides were then placed in a double row in a 260-mm–wide horizontal electrophoresis tank (Consort, Parklaan, Belgium) containing 0.3 mol/L of NaOH and 1 mmol/L of Na_2_EDTA for 40 min before electrophoresis at 25 V for 30 min at an ambient temperature of 4 °C (the temperature of the running buffer not exceeding 15 °C). The slides were then washed three times for 5 min each with 0.4 mol/L of Tris-HCl (Sigma Diagnostics), pH 7.5, at 4 °C before staining with 20 µL of 4 = 6-diamidine-2-phenylindol dihydrochloride (Sigma Diagnostics; 5 µL g/mL). One hundred comets from each gel (scored at random) were scored using a Leica DMLS fluorescence microscope (Leica Microsystems, Wetzlar, Germany) with a computerized image analysis (Komet 5.5; Kinetic Imaging Ltd., Liverpool, United Kingdom).

Power analyses for comet assay have been performed according to the previous calculations made by Smith *et al*.^50^. The number of animals required to obtain 80% power for testicular cells in the present study is comparable to the number of animals required for stomach, liver and bone marrow cells for a 2-, 2.5- and 3-fold change as reported by Smith *et al*.^50^. In the present study, the use of 2 gels per animal compared to 1 gel per animal improved the power noticeably, whereas the impact of a further increase in the number of gels diminished the power. Finally, two gels per animal were used.

### Statistical analysis

Data are reported as mean ± standard error of the mean (SEM). Statistical analyses were performed using SPSS Version 22.0 (SPSS Inc., Chicago, IL, USA). Differences between control and anaemic groups were tested for statistical significance with Student’s t test. One-way analysis of variance (ANOVA) was used to compare both fermented milk-based diets supplied to the both animal groups (control and anaemic). Following a significant F test (P < 0.05), individual means were tested by pairwise comparison with Tukey’s multiple comparison test when main effects and interactions were significant. The level of significance was set at P < 0.05.

## Supplementary information

Supplementary information
