# Publisher Correction: Protective effects of fermented goat milk on genomic stability, oxidative stress and inflammatory signalling in testis during anaemia recovery

**DOI:** 10.1038/s41598-019-42851-1

**Published:** 2019-05-03

**Authors:** Jorge Moreno-Fernandez, María J. M. Alférez, Inmaculada López-Aliaga, Javier Diaz-Castro

**Affiliations:** 1 Department of Physiology, University of Granada, Granada, Spain; 2 Institute of Nutrition and Food Technology “José Mataix Verdú”, University of Granada, Granada, Spain

Correction to: *Scientific Reports* 10.1038/s41598-018-37649-6, published online 19 February 2019

Due to a typesetting error, the original version of this Article was published without vertical delineation in the blots which appear in Figure 2 and in the Supplementary Information file Figure S1. These errors have been corrected in the versions of Figure 2 and the Supplementary Information that now accompany the Article.

