# Supplementary material for: Protective effects of fermented goat milk on genomic stability, oxidative stress and inflammatory signalling in testis during anaemia recovery
Source: Sci Rep. 2019 Feb 19;9:2232. doi: 10.1038/s41598-018-37649-6 (PMC6381118; doi:10.1038/s41598-018-37649-6)
Supplement: Supplementary file 1 — Supplementary information [file 41598_2018_37649_MOESM1_ESM.pdf]

Supplementary information

Protective effects of fermented goat milk on genomic stability, oxidative stress and inflammatory signalling in testis during anaemia recovery

Jorge Moreno-Fernandez[1,2], María JM Alférez[1,2], Inmaculada López-Aliaga[1,2,*] and Javier Diaz-Castro[1,2]

1Department of Physiology, University of Granada, Granada, Spain.

2Institute of Nutrition and Food Technology "José Mataix Verdú", University of Granada, Granada, Spain.

Contents

- Supplementary figure full-length blots (S1)

- Specificity of the polyclonal antibody

Moreno-Fernandez et al., Figure S1

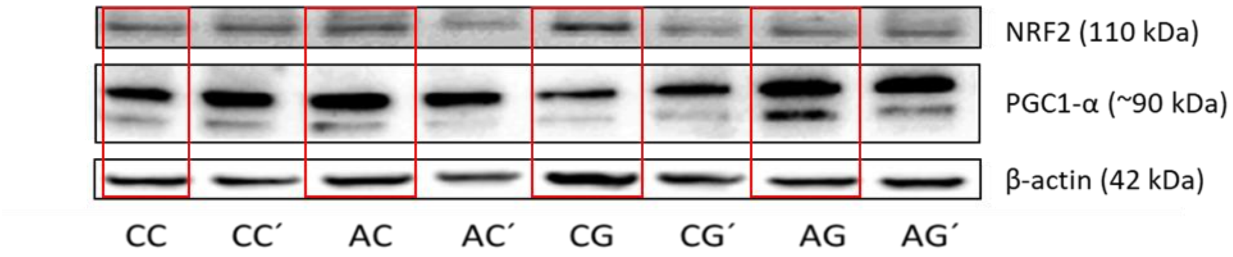

Figure S1. Original full-length blots for the western blots in Figure 2C. Representative blots have been boxed.

Moreno-Fernandez et al., Specificity of polyclonal antibody

To revise the specificity of the polyclonal antibody please visit the following abcam website which includes a datasheet of the polyclonal antibody in which the reviewer can check that is adequate for our study.

https://www.abcam.com/nrf2-antibody-ab31163.html
